# Reinfection studies of canine echinococcosis and role of dogs in transmission of *Echinococcus multilocularis* in Tibetan communities, Sichuan, China

**DOI:** 10.1017/S0031182013001200

**Published:** 2013-08-28

**Authors:** J. E. MOSS, X. CHEN, T. LI, J. QIU, Q. WANG, P. GIRAUDOUX, A. ITO, P. R. TORGERSON, P. S. CRAIG

**Affiliations:** 1 Cestode Zoonoses Research Group, School of Environment and Life Sciences, University of Salford, Manchester M5 4WT, UK; 2 Institute of Parasitic Diseases, Sichuan Centers for Disease Control and Prevention, Chengdu 610041, Sichuan, China; 3 Chrono-environment Lab, UMR 6249 University of Franche-Comte and CNRS, Besancon, France; 4 Department of Parasitology, Asahikawa Medical University, Asahikawa, Hokkaido 078-8510, Japan; 5 Section of Epidemiology, Vetsuisse Faculty, University of Zurich, Zurich, Switzerland

**Keywords:** *Echinococcus multilocularis*, canine echinococcosis, Tibetan communities

## Abstract

In the eastern Tibetan plateau both human cystic and alveolar echinococcosis (AE) caused by infection with *Echincoccus granulosus* or *Echinococcus multilocularis*, respectively are highly endemic. The domestic dog plays a key role in zoonotic transmission in this region. Our primary objective was to investigate the role of domestic dogs in maintaining transmission of *E. multilocularis* in Shiqu county, Sichuan. A cohort of 281 dogs was followed up over one year after a single treatment with praziquantel followed by re-infection surveillance at 2, 5 and 12 months post-treatment. Faecal samples were tested by an *Echinococcus* genus-specific coproantigen ELISA and two species-specific copro-PCR tests. Total *Echinococcus* coproantigen prevalence in Shiqu at baseline was 21% and 9·6% after 2 months. *E. multilocularis* copro-PCR was positive in 11·2% of dogs before treatment (*vs* 3·6% with *E. granulosus* copro-DNA), 2·9% at 2 months post-treatment, and 0% at 5 month and 12 months. The results suggest that dogs may have the potential to maintain *E. multilocularis* transmission within local pastoral communities, and thus dog dosing could be an effective strategy to reduce transmission of *E. multilocularis* as well as *E. granulosus* in these co-endemic Tibetan communities.

## INTRODUCTION

Echinococcosis is a zoonosis that affects both domestic animals and wildlife populations, and is a severe public health concern in much of western China (Craig, 2006; Wang *et al.* 2008*a*). The infection in humans/livestock/small mammals is caused by larval stages and in definitive hosts by adult stages of tapeworms belonging to the genus *Echinococcus*. On the eastern part of the Tibetan Plateau the two most important of these zoonotic species, *Echincoccus granulosus* (the causative agent of cystic echinococcosis, CE) and *Echinococcus multilocularis* (the causative agent of alveolar echinococcosis, AE), have been shown to be highly endemic and co-endemic over a geographical area >900 000 km^2^ (Giraudoux *et al.* 2013*a*). Mass abdominal ultrasound screening in Tibetan communities revealed very high village prevalence rates of 12·1% for human CE and 14·3% for human AE (Li *et al.* 2005, 2010). Budke *et al.* (2006) estimated that the global disability life years (DALYs) and monetary losses resulting from human and livestock CE were 1 009 662 DALYs and US $763 980 979 respectively, and in China alone accounted for 40% of global DALYs lost to echinococcosis.

Canine and vulpine definitive hosts may play a crucial role in the transmission and dissemination of both these species. In Ganze Tibetan Autonomous Prefecture (Sichuan province), the predominant cycle for *E. granulosus* involves the domestic dog and livestock (sheep, goats, yak) (Yang *et al.* 2009), whilst for *E. multilocularis* the natural cycle appears to be between fox species (primarily the Tibetan fox, *Vulpes ferrilata* but also the red fox *Vulpes vulpes*) and a number of small mammal hosts including microtines and lagomorphs (Raoul *et al.* 2006; Wang *et al.* 2008*a*). Due to the high prevalence of both *E. multilocularis* and *E. granulosus* in Tibetan domestic dogs, for example purge rates of 12 and 8% respectively (Budke *et al.* 2005*a*), it has been hypothesized that domestic dogs are responsible for zoonotic transmission not only of *E. granulosus*, but also for *E. multilocularis* in this locality (Wang *et al.* 2001*a*; Vaniscotte *et al.* 2011). It is not clear however whether dogs could maintain *E. multilocularis* in an independent peri-domestic cycle involving dogs and small mammals, or merely act as a reservoir host for infection from spill-over of an active wildlife cycle involving foxes and small mammals. Previous investigations indicated that ownership of dogs was the most important factor associated with risk of human AE disease in Tibetans (Li *et al.* 2005; Wang *et al.* 2006). Other risk factors included occupation (herdsman were at a greater risk), number of dogs owned (the more dogs, the greater the risk), and playing/contact with dogs (direct contamination by eggs) (Wang *et al.* 2006; Giraudoux *et al.* 2013*a*).

**Fig. 1.**
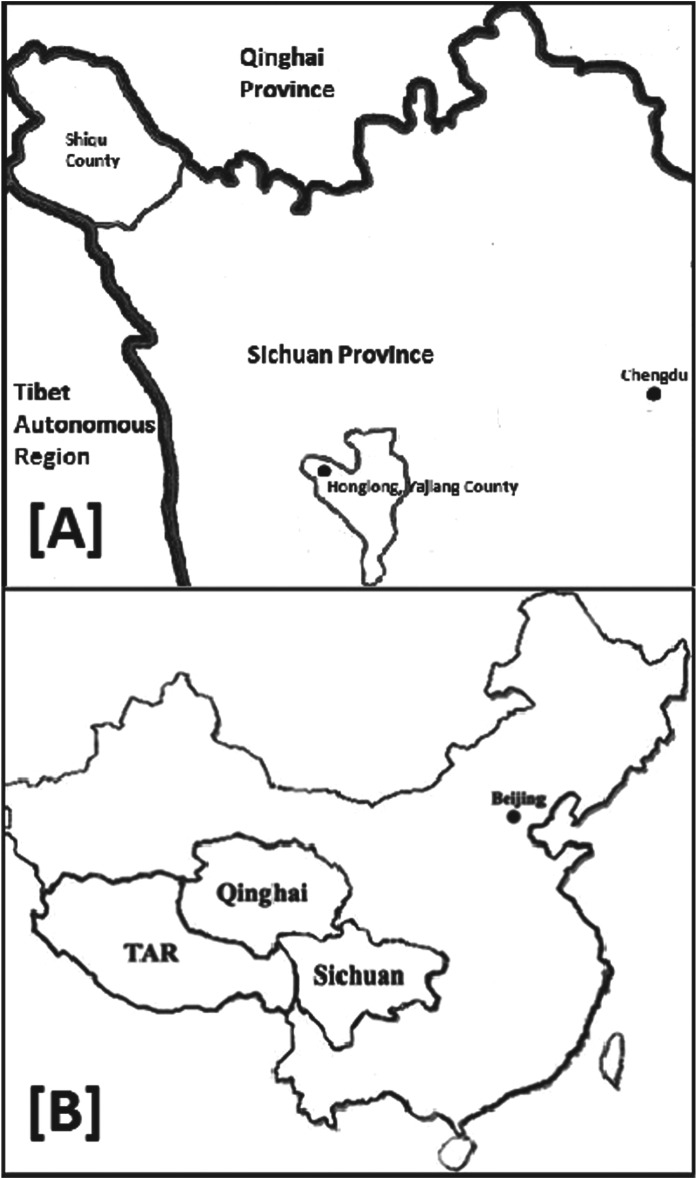
Location of study areas Shiqu and Yajiang counties in Sichuan Province, China.

Theoretically, if domestic dogs transmit *E. multilocularis* to humans then they could also transmit the parasite to small mammals via dog faecal contamination of small mammal habitats close to or surrounding these pastoral communities. If transmission infection to small mammals from dogs occurs at a sustainable level, then infected small mammals could subsequently provide infection pressure to the large and accessible dog population. Prevalence of *E. multilocularis* in small mammal species is typically variable and often below 5%, but in Tibetan areas of Sichuan province prevalence ranging from <5 to >20% have been reported in *Microtus* spp., *Ochotona* spp. and *Lepus* spp. (reviewed by Craig, 2006; Wang *et al.* 2008*a*). Shiqu county, located in the north of Ganze Tibetan Autonomous Prefecture, is reported as a highly endemic region for both human CE and AE, whereas other parts of Ganze Prefecture have much lower human prevalence rates. In Honglong (Yajiang county) in the south of Ganze Prefecture (Fig. 1), a total of 610 people were screened for AE and CE, while there were no human AE cases reported in the area and the human CE prevalence was relatively low at 2·3%, compared to 6·2% prevalence of human CE in Shiqu county (Li *et al.* 2010). Nevertheless, Honglong is similar to Shiqu in terms of landscape characteristics potential habitats for Tibetan foxes and small mammal species.

Monitoring the level of echinococcosis in human and animal populations is essential to the understanding of epidemiology, control and prevention of transmission (WHO/OIE, 2001). Mathematical models which describe transmission dynamics have also proved useful to assess and predict outcomes of intervention studies (Budke *et al.* 2005*b*; Torgerson, 2006). One parameter for epidemiological studies is the rate at which dogs naturally become infected or re-infected by *Echinococcus* species. Previous studies in endemic CE regions have attempted to estimate the natural re-infection of *E. granulosus* in dogs e.g. in northern Kenya (Wachira *et al.* 1990), Uruguay (Cabrera *et al.* 1996), south Argentina (Larrieu *et al.* 2000), northern Libya (Buishi *et al.* 2005), Tunisia (Lahmar *et al.* 2008) and northwest Xinjiang, China (Wang *et al.* 2001*a*, *b*). No similar reinfection studies for *E. multilocularis* in dogs have as yet been reported.

In the current study, we have tried to assess the natural re-infection of *E. multilocularis* (and *E. granulosus*) in dogs from a highly co-endemic Tibetan pastoral region (Shiqu county, Sichuan, China). Furthermore the potential role of dogs in the sustainable transmission of *E. multilocularis* is considered.

## APPROACH AND METHODS

Two study locations were chosen based on the availability of previous data for both human and animal echinococcosis infection rates. The main site was Shiqu county located in the northwest of Ganze Tibetan Autonomous Prefecture (Sichuan) on the south-eastern fringe of the Qinghai-Tibet Plateau, at the meeting point of Qinghai, Sichuan and Tibet. The whole county sits at an altitude of approximately 4200 m and covers an area of 25 141 km^2^ of which about 19 000 km^2^ (90%) of this county is classified as grazing pasture. Average annual temperature is −1·6 °C, with yearly rainfall ranging from 460 to 636 mm. Ethnic Tibetans make up 98% of the population (total 71 000) of Shiqu (Wang *et al.* 2006). A second site investigated was Honglong located in Yajiang county approximately 570 km south of Shiqu county but still within Ganze Tibetan Autonomous Prefecture. The altitude in Honglong region is approximately 4100–4500 m with almost identical landscape and pastoral features to Shiqu.

Owned dogs were registered, with owner permission, for a reinfection study in eight village clusters of Shiqu county. The position of each dog was recorded via a hand held GPS (GPS60, Garmin International Inc, USA). Dog faecal samples were collected from tied dogs at four time points over the period 2006–2008. Two ground faecal samples from each dog were collected (one for DNA analysis, one for coproantigen analysis) at baseline or time ‘0’. Re-sampling occurred at 2 month post treatment (mpt) with praziquantel, 5 and 12 mpt. A single dose of praziquantel was administered orally to dogs at the recommended dose of 5 mg kg^−1^ (reported 99·9% efficacy) (WHO/OIE, 2001) under supervision in order to eliminate any tapeworms at the start of the study (time 0) in the Shiqu communities. All faecal samples were tested using an established coproantigen ELISA (Allan *et al.* 1992) for the detection of *Echinococcus* genus-specific faecal antigens (>98% genus specific) and also subjected to PCR. Total DNA was extracted from 2 g faeces using the QIAamp^®^ DNA Stool Mini Kit (Qiagen, UK) and *Echinococcus* spp. DNA analysed using two copro-PCR tests for the species specific detection of *E. multilocularis* and *E. granulosus* DNA based on the ND1 gene (Boufana *et al.* 2013).

Ethical approval was obtained from the Sichuan Centre for Disease Control (CDC), Sichuan, PR China. Dog owners participated in the study voluntarily and were informed of all aspects of the research prior to cooperation. To obtain infection data in tethered owned dogs, all available village dogs were sampled via the collection of ground faeces associated with a specific dog. In Shiqu participants also completed a dog owner's questionnaire designed to obtain information on demographics (age, sex, occupation), animal ownership, husbandry practices and dog behaviour. In Honglong community, only the age and sex of dogs were noted, and dogs were not followed up after baseline sampling. Risk factors associated with canine echinococcosis, as identified by positive copro-ELISA and copro-PCR, were evaluated statistically. Data sets were analysed using SPSS 17.0 for windows (IBM, Chicago, ILL). Prevalence rates between locations were investigated using Fisher's exact test. Results of statistical tests were classed as significant at a *P*<0·05 level.

## RESULTS

### Baseline prevalence in dogs

**Fig. 2.**
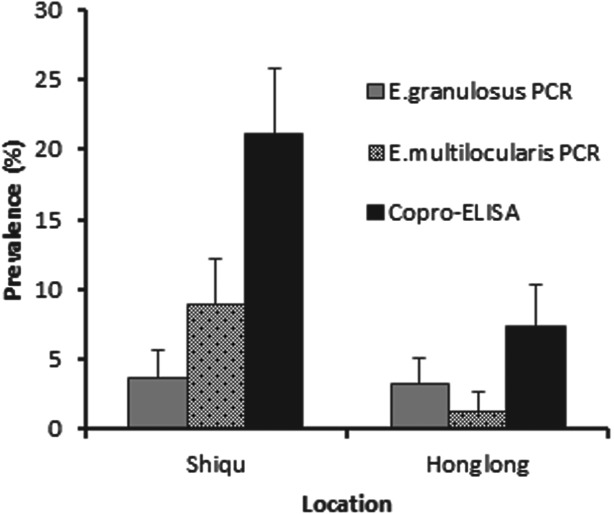
Total copro-prevalence of echinococcosis in dogs from Shiqu and Honglong sites. Dogs were tested by coproantigen ELISA and copro-PCR at baseline (before deworming).

**Fig. 3.**
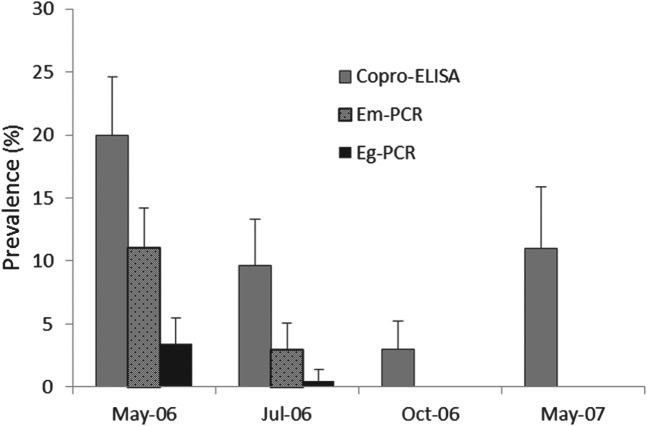
Copro-prevalence of *E. multilocularis* and *E. granulosus* in owned dogs from Shiqu county tested by species specific copro-PCR. Copro-ELISA *Echinococcus* spp. prevalences are also given. Dogs were treated once with praziquantel (in May 2006) and tested at 2 months (July 2006), 5 months (October 2006) and 12 months (May 2007) after deworming.

A total of 592 dogs were registered for an *Echinococcus* prevalence study between May 2006 and May 2007. There was a higher proportion of male to female dogs at a ratio of 3·2 : 1. Baseline total prevalence of *Echinococcus* species in owned dogs from Ganze prefecture, determined by combining results from both copro-ELISA and copro-PCR assays was 27·2% (CI±5·2%) in Shiqu county, *vs* 10·6% (CI±3·4%) in Honglong (Yajiang county), a highly significant difference (*P*⩽0·001) (Fig. 2). In total, 21% of dogs in the Shiqu sample were coproantigen positive at baseline. The number of dogs which tested positive for *Echinococcus* spp. by the coproantigen ELISA was greater than those tested positive by the copro-PCR. There were 58 dogs out of 276 (21%) (five samples were spoiled) which were copro-ELISA positive in Shiqu, of these 10 (3·6%) were *E. granulosus* PCR positive, and 31(11·2%) *E. multilocularis* PCR positive out of 276 dogs (Fig. 3). Furthermore, 1 sample was *E. granulosus* copro-PCR positive/coproantigen negative and 19 samples were *E. multilocularis* copro-PCR positive/coproantigen negative. In Honglong, a low risk area for human AE, 23 out of 311 dogs (7·4%) were *Echinococcus* copro-ELISA positive, with a total of 14 samples being positive by PCR (4 *E. multilocularis* and 10 *E. granulosus*). Statistical analysis (Fisher's exact test) revealed that according to the coproantigen ELISA there was a significant difference in prevalence between Shiqu and Honglong areas (*P*⩽0·001). There was no significant difference between the prevalence of *E. granulosus* at the two study locations (Shiqu *vs* Honglong) when tested with the specific copro-PCR (Fisher's exact test, exact *P* values = 0·824–1·00). When the samples were tested with the *E. multilocularis* species-specific copro-PCR test there was a significantly higher prevalence in Shiqu (8·9%) (*P*⩽0·001) compared to Honglong (1·3%) (Fig. 2).

### Reinfection study in dogs by coproantigen ELISA

In May 2006 (baseline, time ‘0’), a total cohort of 281 owned dogs were registered (sampled and dosed once with praziquantel) from eight village clusters in Shiqu county to establish a re-infection rate of *E. granulosus* and *E. multilocularis* within the dog population over 1 year. Dogs between the ages of 3 and 6 years were the most numerous in the population with a mean age at 4·2 years. According to their Tibetan owners, dogs were kept for the purpose of guarding the household and livestock. Male dogs appeared to be better for this role; the larger and more aggressive the dog, the more pride was taken in that animal.

The number of surveyed dogs decreased throughout the 12 month study period due to loss, death or absence from study locations (i.e. dogs were accompanying herders). In July 2006, 2 months post-treatment (mpt), 72·9% of the original dog population (compared to baseline, in May 2006) was sampled. In October 2006, this decreased to 71·2% and at 12 mpt May 2007, just over half (54·8%) of the registered dogs could be successfully re-sampled. A total of 106 dogs (37·7%) (= *individual cohort*) of the original 281 registered dogs (= *total cohort*) were re-sampled at every time point. Results obtained from the individual cohort were analysed as part of the whole dog population and also as a separate cohort.

The baseline prevalence of *Echinococcus* spp. in owned dogs, as assessed by the coproantigen ELISA, in May 2006 was 21% [CI±4·6%] (Fig. 3). Dogs were given a single dose of praziquantel in May 2006 and two months later in July 2006 (∼65 days) the dogs were re-sampled, wherein they displayed a coproantigen prevalence of 9·6% [CI±3·7%] within the total dog cohort population (or 11·3% in the individual dog cohort followed each time). This equated to an *Echinococcus* spp. re-infection rate of 45·7% (9·6/21 i.e. proportion of dogs infected compared to baseline) after 2 months for the total cohort (or 57% in the individual cohort where the same individual dogs were re-tested). The copro-prevalence in October 2006 was 3·1% [CI±2·2%] in the total dog cohort, and 2·8% [CI±3·2] in the individual dog cohort. This suggests that the overall *Echinococcus* spp. re-infection rate after 5 months was 15·5%. By May 2007, a year after a single dose of praziquantel, total echinococcosis copro-prevalence had increased to 11% [CI ±4·9%] for the total cohort (Fig. 3), and to 12·3% for the individual dog cohort (i.e. same dog sampled at each time point).

There was a significant difference (i.e. reduction) in *Echinococcus* copro-prevalence between May 2006 and July 2006 (*P*⩽0·001), and between July 2006 and October 2006 (*P*⩽0·05). In contrast, a significant increase in total copro-prevalence occurred between July 2006 and May 2007 (*P*⩽0·05). There was also a significant difference between the prevalence in May 2006 and May 2007, a year after praziquantel treatment (*P*⩽0·05). These differences were uniform in both the total and individual dog cohorts.

### Reinfection study in dogs by copro-PCR

*Echinococcus* species re-infection of dogs by either *E. multilocularis* or *E. granulosus* was investigated by copro-PCR testing of all faecal samples using species specific ND1 primers described by Boufana *et al.* (2013). The number of positive copro-ELISA (i.e. genus *Echinococcus* spp.) tests was far greater than the number generated by the copro-PCR assays. Whilst 58/276 (21%) samples were copro-ELISA positive at the baseline in May 2006, 31 were *E. multilocularis* PCR positive (11·2%) and 10 *E. granulosus* PCR positive (3·6%) (Fig. 3). Within this group, seven samples tested positive by both the copro-ELISA and *E. multilocularis* PCR and nine tested positive in both. In July 2006 (2 mpt), six (2·9%) of the dog faecal samples were *E. multilocularis* PCR positive, and 1 (0·5%) was *E. granulosus* PCR positive out of 205. No dog was copro-PCR positive at 5 mpt (October 2006) or 12 mpt (May 2007).

### Township/village level results

**Fig. 4.**
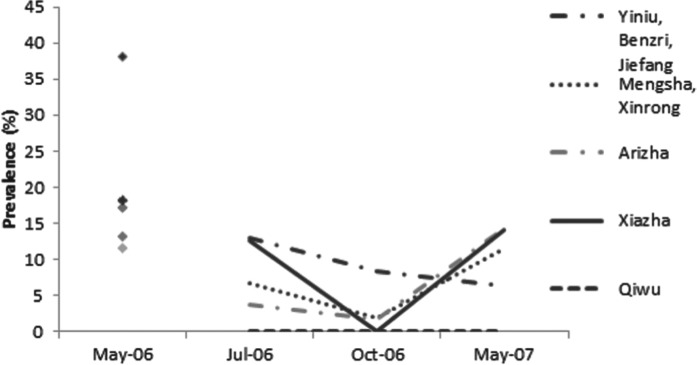
Copro-prevalence of *Echinococcus* spp. in dogs followed-up using copro-ELISA after deworming in May 2006 from five townships in Shiqu county, Sichuan. Baseline in May 2006 (total dogs *n* = 308 dogs), 2 months post treatment in July 2006 (*n* = 205), 5 months post treatment in October 2006 (*n* = 199) and 12 months post treatment in May 2007 (*n* = 163). For May 2006 symbols top = Yiniu, 2nd = Mengsha, 3rd = Xiazha, 4th = Qiwu, bottom = Arizha.

At the township/village level, the trends in infection and re-infection prevalence as assessed by copro-ELISA varied slightly from place to place (Fig. 4). The highest baseline coproantigen prevalence was found in the township of Yiniu (i.e. Yiniu/Benzri/Jiefang cluster, *n* = 68) at 38% [CI±12%] (24 out of 63 were positive by copro-ELISA). After 65 days, the re-infection prevalence was 13% (6 out of 45), suggesting a rapid re-infection rate of 34% (i.e. 13 *vs* 38%). In Mengsha (Mengsha/Xinrong cluster, *n* = 55), Arizha (*n* = 69) and Xiazha (*n* = 89) communities, the re-infection prevalence followed a trend similar to the total dog population. In May 2007 the prevalence in dogs from these townships showed no difference from that of May 2006 (*P*⩽0·05 in each location). In the most distant township in south Shiqu county (i.e. Qiwu), the baseline copro-prevalence was 13% but dogs did not appear to become re-infected based on copro-ELISA data as all copro-ELISA test results remained negative for the 12 month post treatment follow-up.

### Questionnaire analysis

The results of the coproantigen ELISA and copro-PCR (*E. granulosus* and *E. multilocularis*) tests were examined with reference to questionnaire responses. Only one of the questions – ‘when is your dog tied?’- (of the following: occupation, livestock ownership, hunt foxes, echinococcosis case in family, dog consumes raw meat, dog consumes small mammals, when is dog tied) gave a significant result. Twenty-five out of 165 (15·2%) dogs which were always tied were positive by copro-ELISA, whilst 33 out of 109 (30·3%) dogs which were tied in the day only, were positive by the copro-ELISA, the latter being significantly higher (*P*⩽0·001).

## DISCUSSION

China has the highest recorded prevalence of human AE in the world (Torgerson *et al.* 2010) as well as the one of the highest levels of human CE (Li *et al.* 2005; Budke *et al.* 2006). The prevalence of human echinococcosis (CE and AE) in Tibetan pastoral communities in Sichuan Province at prefecture and county levels does vary greatly (<0·5% to >10%) with some areas co-endemic and others where either CE or AE cases predominate (Li *et al.* 2010; Giraudoux *et al.* 2013*a*). The latency time for disease diagnosis/clinical manifestation in humans ranges from 5–15 years, with generally a greater asymptomatic period for AE cases, and thus less probability of early hospital treatment (Yang *et al.* 2006).

Shiqu county in Ganze Tibetan Autonomous Prefecture (Sichuan) is reported as a highly endemic region for both human CE and AE with mean ultrasound prevalences of 6·5 and 6% respectively, whereas other parts of Ganze Prefecture have much lower human prevalence rates (Li *et al.* 2010). In Honglong (south Ganze) for example, CE was recorded from mass ultrasound surveys at 2·3% and no cases of AE were detected (Li *et al.* 2010). The prevalence of *E. multilocularis* and *E. granulosus* in domestic dogs after purgation was recorded at 12 and 8%, respectively in Shiqu (north Ganze) (Budke *et al.* 2005*a*). It was hypothesized that domestic dogs are responsible for the transmission, not only of *E. granulosus*, but also for *E. multilocularis* in this locality (Wang *et al.* 2001*a*, 2006; Vaniscotte *et al.* 2011). It is not clear, however, whether dogs could maintain *E. multilocularis* in a fox-independent transmission cycle between dogs and small mammals in the region, or if dogs merely act as a reservoir host for infection from spill-over of a fox-small mammal wildlife cycle.

A one year follow-up study (2, 5 and 12 month sampling) was undertaken in a cohort of owned dogs in Shiqu (a highly endemic AE area) to assess the outcome of intervention from a single dosing round with praziquantel, and to measure the natural re-infection rate of *E. multilocularis*. A low endemic human AE area with similar landscape and community features (Honglong) was used as a baseline control/comparison site. It was determined that in Shiqu county dogs were infected with *Echinococcus* species at a significantly higher total copro-prevalence (27·2%) than Honglong (10·6%). However, results of the copro-PCR analysis revealed the copro-prevalence of *E. granulosus* DNA in dogs was the same for both localities (∼3%), and thus the baseline presence of *E. multilocularis* in dogs (11·2%) in Shiqu was more likely the cause of this difference. By 2 months after dosing, 2·9% of dogs in Shiqu were infected (re-infected) with *E. multilocularis* based on copro-PCR data, compared to 0·5% with *E. granulosus*. At 5 and 12 months post treatment points no dogs were copro-PCR positive for either *E. multilocularis* or *E. granulosus* DNA. In contrast, coproantigen prevalence (*Echinococcus* genus specific) was 3% at 5 months post-treatment and 11% one year after dogs were dosed. This difference may be due to the presence of immature worms at 5 and 12 months and/or a lack of sensitivity for the copro-PCR tests.

The period between supervised dog dosing with praziquantel and the first follow-up sampling was 65 days, and as the drug efficacy is >99% any test-positive dogs were thus assumed to be new infections. This period allows sufficient time for both *E. granulosus* and *E. multilocularis* species to become established and reach patency. *E. granulosus* has a pre-patency period of approximately 45 days in dogs (Kumaratilake *et al.* 1983), whereas *E. multilocularis* has a pre-patent time of approximately 30 days in experimentally infected dogs (Kapel *et al.* 2006). Since the life-span of adult tapeworms of *E. multilocularis* is described as either 3 (Kapel *et al.* 2006) or 5 months (WHO/OIE, 2001) compared with 10 months for *E. granulosus* (Aminjanov, 1975), it is possible that any *E. multilocularis* worms established in the gut could produce eggs and subsequently perish (i.e. natural loss of infection) within 5 months post-treatment.

The current findings suggest that the infection pressure to dogs from *E. multilocularis* (in the small mammal reservoir) was higher than that from *E. granulosus* (from the livestock reservoir), an outcome which supports previous work by Budke *et al.* (2005*b*). That study made use of worm abundance data gathered from arecoline purgation to describe the infection pressure of both *Echinococcus* species to dogs in Shiqu county, and they showed that the owned dog population was infected with *E. granulosus* at a mean rate of 0·21 infections a year, and with *E. multilocularis* at a mean of 0·85 infections per year (assuming a 3 month life-span) (Budke *et al.* 2005*b*). It is also indicated from epidemiological and experimental infections that little or no immunity occurs in dogs against *E. multilocularis* infection, in contrast to red foxes, thus dogs of all ages may be susceptible (Budke *et al.* 2005*b*; Kapel *et al.* 2006).

The prevalence of *Echinococcus* coproantigen at village level ranged between 38% (for Yiniu) to 11·6% (for Arizha) at baseline. In all sites (apart from Qiwu) the re-infection prevalence was highest in the first follow-up sampling period (65 days post treatment), suggesting a high *Echinococcus* spp. infection pressure to dogs in the late spring and early summer months. In Yiniu Township, the copro-ELISA re-infection prevalence was 13% indicating a rapid re-infection rate of 34% by July 2006 (after 65 days). In October 2006 and then May 2007, the copro-prevalence decreased indicating that dogs had naturally lost their infections. The copro-ELISA re-infection prevalence was 22% by October and 16·6% by May 2007 (at 12 months post-treatment). The fact that the prevalence was significantly lower in May 2007 compared to May 2006 could indicate that the infection pressure to dogs had reduced in this short period (and thus the risk to humans had also reduced). In the Yiniu township village cluster (Yiniu, Jiefang, Benri), two dogs were PCR test positive for *E. multilocularis* at the first follow-up time (2 months post-treatment). The same two dogs were PCR DNA negative (in absence of deworming) by October 2006 (5 months post-treatment). This supports the observation of natural loss of *E. multilocularis* infection over 3–5 months in experimentally infected dogs (Kapel *et al.* 2006). The absence of *E. multilocularis* or *E. granulosus* PCR-positive dogs (i.e. eggs in faeces) in the October 2006 and May 2007 sample cohorts is surprising, but suggests that worm burdens were low and/or any adult worms present were predominantly pre-patent. For *E. multilocularis* this result might also reflect reduced winter/early spring predation by dogs on small mammals.

The current study shows that the only significant risk factor for an owned dog to test copro-positive for *Echinococcus* spp. in Shiqu, was the owner releasing their dog(s) (from tether) at night (*P* = 0·004). Releasing dogs to free-roam allows them to move around villages and their surroundings at night and presumably have more chance to consume or scavenge nocturnally on active small mammals. Vaniscotte *et al.* (2011) used GPS collars to show that the average home range of owned dogs in Shiqu was 2·6 ha and dogs generally stayed within 115 m from their home-base, though maximum range was 1500 m. Free-roaming occurrence was also significantly associated with *E. multilocularis* infection in dogs based on an arecoline purgation study in the same locality (Budke *et al.* 2005*a*). In these Tibetan pastoral communities, the main *E. multilocularis* biomass is probably mostly in domestic dogs and the small mammal intermediate host population. Although the density of Tibetan foxes (*V. ferrilata*) in Shiqu County is significant i.e. 72 active dens over 230 km^2^ (Wang *et al.* 2008*b*), it is relatively low compared to the dog population estimated to be approximately 30 000 including >4000 strays (Budke *et al.* 2005*c*). Small mammal potential hosts include microtine rodents (e.g. *Microtus limnophilus, M. irene, M. fuscus*), the Kam dwarf hamster (*Cricetulus kamensis*) and the plateau pika (*Ochotona curzoniae*) (Wang *et al.* 2004, 2010). Importantly, *Microtus* and *Cricetulus* species are to some extent both diurnal (or crepuscular) and nocturnal (Smith and Xie, 2008). This means that dogs may have an equal opportunity to prey upon those species if released at night or during certain dusk and dawn periods. Whereas the pika, *O. curzoniae*, for example is diurnal so that most owned dogs (which are usually tethered during the day) may not be free to predate pika during the day. Stray dogs would however have access to predate small mammals at day and night.

Further evidence for the potential of dogs to maintain an independent *E. multilocularis* transmission cycle comes from experimental studies in definitive hosts. When compared with red foxes, dogs were shown to maintain higher and more consistent worm burdens over a 90 day period (Kapel *et al.* 2006). These results strongly indicate that dogs are good hosts for *E. multilocularis* and, in Shiqu where dog-small mammal contact is high, they are more likely to represent a transmission threat given their sustained worm burden, low resistance and biotic potential. The role of Tibetan foxes in maintaining a wildlife cycle of *E. multilocularis* is also evident from recent studies in west Shiqu County which demonstrated a copro-PCR prevalence of 27% (32/74) (Jiang *et al.* 2012). Thus a combination of wildlife and peri-domestic cycles for *E. multilocularis* may occur in/near townships with both high dog and fox populations.

In summary, the current results based on the reinfection profile of dogs from Shiqu after a single deworming treatment with praziquantel, suggest that a proportion of Tibetan dogs quickly became reinfected by summer with *E. multilocularis* but worms were probably lost by 5 months post-treatment (in the Autumn) and that relatively little re-exposure occurred by 12 months. This suggests that the biomass of *E. multilocularis* in owned dogs is important for egg contamination of the environment and subsequent exposure of small mammal hosts. This further supports the possibility that dogs contribute to an active peri-domestic cycle for *E. multilocularis*. Finally, although further dog cohort follow-up studies are required over a more intensive intervention programme in order to determine more precisely the role of dogs in sustainable transmission of *E. multilocularis*, the results from this study indicate that a single round of supervised praziquantel dosing of owned dogs can substantially reduce prevalence of infection of *E. multilocularis* in dogs. Therefore an increased dosing frequency of 2–3 times per year would probably have a major impact on both zoonotic risk and transmission potential for both *E. multilocularis* and *E. granulosus* in these co-endemic Tibetan communities.
